# *Vibrio japonicus* sp. nov., a novel member of the Nereis clade in the genus *Vibrio* isolated from the coast of Japan

**DOI:** 10.1371/journal.pone.0172164

**Published:** 2017-02-23

**Authors:** Hiroyasu Doi, Ikuko Osawa, Hayamitsu Adachi, Manabu Kawada

**Affiliations:** 1 Institute of Microbial Chemistry (BIKAKEN), Numazu, Numazu-shi, Shizuoka, Japan; 2 Institute of Microbial Chemistry (BIKAKEN), Shinagawa, Tokyo, Japan; Beijing Institute of Microbiology and Epidemiology, CHINA

## Abstract

A novel *Vibrio* strain, JCM 31412^T^, was isolated from seawater collected from the Inland Sea (Setonaikai), Japan, and characterized as a Gram-negative, oxidase-positive, catalase-negative, facultatively anaerobic, motile, ovoid-shaped bacterium with one polar flagellum. Based on 16S rDNA gene identity, strain JCM 31412^T^ showed a close relationship with type strains of *Vibrio brasiliensis* (LMG 20546^T^, 98.2% identity), *V*. *harveyi* (NBRC 15634^T^, 98.2%), *V*. *caribbeanicus* (ATCC BAA-2122^T^, 97.8%) and *V*. *proteolyticus* (NBRC 13287^T^, 97.8%). The G+C content of strain JCM 31412^T^ DNA was 46.8%. Multi-locus sequence analysis (MLSA) of eight loci (*ftsZ*, *gapA*, *gyrB*, *mreB*, *pyrH*, *recA*, *rpoA* and *topA*; 5535bp) further clustered strain JCM 31412^T^ in the Nereis clade, genus *Vibrio*. Phenotypically, strain JCM 31412^T^ differed from the closest related *Vibrio* species in its utilization of melibiose and raffinose, and its lack of casein and gelatin hydrolysis. It was further differentiated based on its fatty acid composition, specifically properties of C_12:0_3OH and summed features, which were significantly different from those of *V*. *brasiliensis*, *V*. *nigripulchritudo* and *V*. *caribbeanicus* type strains. Overall, the results of DNA-DNA hybridization, and physiological and biochemical analysis differentiated strain JCM 31412^T^ from other described species of the genus *Vibrio*. Based on these polyphasic taxonomic findings, it was therefore concluded that JCM 31412^T^ was a novel *Vibrio* species, for which the name *Vibrio japonicus* sp. nov. was proposed, with JCM 31412^T^ (= LMG 29636^T^ = ATCC TSD-62^T^) as the type strain.

## Introduction

The family *Vibrionaceae*, in class Gammaproteobacteria, currently includes nine recognized genera: *Vibrio* [1], *Photobacterium* [2], *Catenococcus* [3], *Grimontia* [4], *Echinimonas* [5], *Salinivibrio* [6], *Enterovibrio* [7] and *Aliivibrio* [8]. To date, 131 species of *Vibrio* including two subspecies have been described (www.bacterio.net/vibrio/html). Vibrios were further grouped into 16 clades by multi-locus sequence analysis (MLSA) [9, 10]. Most have not been implicated in disease; however, *V*. *cholerae*, *V*. *parahaemolyticus* and *V*. *vulnificus* are commonly identified as causative agents of human. In general, *Vibrio* species are halophilic, mesophilic or chemoorganotrophic in nature, with facultatively fermentative metabolism [11]. They are ubiquitous inhabitants of aquatic environments including marine, coastal, freshwater and sediments, as well as being eukaryotic hosts [12–14]. In this paper, we describe a new species (strain JCM 31412^T^) isolated from seawater from the Inland Sea, Japan, for which the name *Vibrio japonicus* is proposed.

## Materials and methods

### Bacterial strains and growth conditions

The type strain *Vibrio japonicus* JCM 31412^T^ was isolated from surface seawater (26°C) of the Inland Sea (Setonaikai), Ako City, Hyogo Prefecture, Japan (latitude 34°44ʹ44ʺN, longitude 134°22ʹ35ʺE) in Oct. 2013. Strain JCM 31412^T^ was isolated with an agar medium that contained 0.5% Bacto peptone (Difco), 0.1% Bacto yeast extract (Difco), 0.01% Iron (III) phosphate and 1.5% agar (Difco) in artificial seawater (Osaka Yakken) (pH 7.6). Stock cultures were frozen at -80°C in 20% glycerol (w/w) until use. Strains were grown aerobically on tryptone soy agar (TSA; Oxioid) supplemented with 2% (w/v) NaCl for 24h at 28°C. Strain JCM 31412^T^ was deposited in the Japan Collection of Microorganisms (JCM), Microbe Division, RIKEN BioResource Center, Ibaraki, Japan, the BCCM/LMG Bacteria Collection at Ghent University (Belgian Coordinated Collection of Microorganisms), Gent, Belgium and the American Type Culture Collection (ATCC), Manassas, VA, USA.

### Phenotypic characterization

Strain JCM 31412^T^ was subjected to the following phenotypic tests: cell morphology and mobility; Gram staining; Voges-Proskaur test; oxidase and catalase activity; oxidation/fermentation; hydrolysis of gelatin, esculin and casein; and DNA decomposition. Catalase activity was determined by bubble formation in 3% H_2_O_2_ solution, and oxidase activity using cytochrome oxidase paper (Nissui, Tokyo). DNA decomposition (DNase production) was tested by using the toluidine blue-DNA agar containing 1.0% Bacto peptone (Difco), 1.0% yeast extract (Difco), 0.2% deoxyribonucleic acid (from salmon testes), 0.01% toluidine blue, 2.0% agar (Difco) and 3.0% NaCl (pH 7.6). Growth at various temperatures (4–40°C), pH (4–12), NaCl concentrations (0–10%) using tryptic soy broth (Difco) with 1.5% NaCl (for temperature and pH) at 28°C (for pH and NaCl) with shaking, and on thiosulfate-citrate-bile sucrose (TCBS) agar (Nippon Beckton Dickinson) was also examined. Additional biochemical characterization was performed using standardized API 20E, API 20NE, API ZYM and API 50CH identification systems (bioMérieux) with incubation at 28°C according to the manufacturers’ instructions, except that sterile 1.5% (w/v) NaCl was used to prepare the inocula. Strain JCM 31412^T^ was phenotypically characterized in comparison with the following closely related *Vibrio* species obtained from bacterial culture collections: *V*. *brasiliensis* LMG 20546^T^ [15], *V*. *nigripulchritudo* LMG 03896^T^ [16] and *V*. *caribbeanicus* ATCC BAA-2122^T^ [17] (Table 1). Antibiotic sensitivity was established using a disc susceptibility assay as described by the Clinical and Laboratory Standard Institute (CLSI), document M45-A2 (2010) [18]. Sensitivity to the vibriostatic agent O/129 (2, 4-diamino-6, 7-diisopropylpteridine) was determined using Oxoid discs containing 10 and 150μg O/129 per disc. The inhibition zone of each antibiotic was measured using strains grown on Müller-Hinton agar (Difco) supplemented with 1.5% (w/v) NaCl for 16-18h at 35°C. For negatively stained transmission electric micrograph observations of strain JCM 31412^T^ cells, cells from an overnight culture in tryptic soy broth (Difco) with 1.5% NaCl at 28°C with shaking were harvested by centrifugation and washed with sterile saline, and were then fixed for 30 min at 4°C in 2% glutaraldehyde in 0.1M phosphate buffer (pH 7). A droplet of cell suspension was placed on carbon-film with a 400 Cu grid for two minutes. Excess liquid was removed using filter paper. The cells on the grid were negatively stained with 2% (w/v) uranyl acetate (Cerac, USA) for two minutes after drying the excess liquid at room temperature. Samples were observed by transmission electron microscopy (TEM) with H-7600 (HITACHI, Tokyo) at 100 kV.

**Table 1:** Phenotypic characteristics differentiating *Vibrio* sp. nov. JCM 31412^T^ from type strains of phylogenetically related *Vibrio* species.

Characteristic	1	2	3	4
**Growth in/at:**				
Temperature (°C)	10–37	20–37	20–35	20–30
pH	7–12	6-12-	6–10	6–9
NaCl (%)	0.5–9.0	0.5–7.0	0.85–6	0.5–5
**OF-test**	F	F	F	O
**Fermentation of:**				
sucrose	+	+	-	-
mannitol	+	+	-	-
melibiose	+	+	+	-
D-amygdalin	+	+	+	-
**Utilization of:**				
mannose	-	+	+	-
raffinose	+	-	-	-
pyruvic acid-Na	-	-	-	+
citric acid	+	+	+	-
L-tryptophan	+	+	+	+
**Hydrolysis of:**				
gelatine	-	+	+	+
esculin	-	+	+	-
casein	-	+	+	+
DNA	+	+	+	-
**Growth on:**				
TCBS medium	+	+	-	-
**Produce of:**				
α-galactosidase	+	-	-	-
β-galactosidase	-	+	+	-
α-glucosidase	-	+	+	+
β-glucronidase	+	-	-	-
lipase (C14)	-	+	+	+
cystinarylamydase	-	-	+	+
trypsin	-	-	-	+
acidic phosphatase	-	-	+	+
amigdaline	+	+	+	-
arginine dehydrolase	+	+	-	-
lysine decarboxylase	+	+	-	-
ornithine decarboxylase	-	-	+	-

Strains: 1, *Vibrio* sp. nov. JCM 31412^T^; 2, *Vibrio brasiliensis* LMG 20546^T^; 3, *Vibrio nigripulchritudo* LMG 03896^T^; 4, *Vibrio caribbeanicus* ATCC BAA-2122^T^. All data were obtained in this study.

+: positive, -: negative, F: fermentative, O: oxidative.

### Fatty acid analysis

Cellular fatty acid methyl esters were obtained from strain JCM 31412^T^, *Vibrio brasiliensis* LMG 20546^T^, *V*. *nigripulchritudo* LMG 03896^T^ and *V*. *caribbeanicus* ATCC BAA-2122^T^ by saponification, methylation and extraction after growth for 24h at 28°C on trypticase soy agar containing 1.5% NaCl (w/v). The cellular fatty acids were extracted according to the protocol of the Sherlock Microbial Identification System [19] and subsequently identified by gas chromatography. Individual fatty acids were identified using the Microbial Identification software package (Sherlock version 6.0) based on the TSBA6 calculation method and TSBA6 library databases [20]. Fatty acid methyl esters were analyzed by gas chromatography with flame-ionization detection (GC-FID) using rapid Microbial Identification System software (RBTR20; MIDI Inc.) to identify the relative amounts of each fatty acid. For comparison, the three closest species based on 16S rDNA analysis were also examined in parallel.

### DNA isolation and genotypic analysis

Genomic DNA was extracted and purified using a commercial QIAamp DNA Blood Maxi Kit (QIAGEN) following the manufacturer’s protocol. The base sequence of complete 16S rDNA was determined according to the method of Nakagawa et al. [21]. Sequences were analyzed with an ABI PRISM 3130 xl genetic analyzer system (Applied Biosystems, CA, USA). A homology search was carried out using the BLASTN (https://blast.ncbi.nlm.nih.gov) and the 16S rDNA base sequences of other known related strains were retrieved from GenBank/EMBL/DDBJ. Sequences were aligned using CLUSTAL W [22] and phylogenetic trees reconstructed using neighbor-joining algorithms (MEGA version 5.05) [23, 24]. The stability of the grouping was estimated by bootstrap analysis with 1000 replications. Multi locus sequence analysis (MLSA) of eight concatenated housekeeping gene sequences encoding topoisomerase I (*topA*), cell division protein (*ftsZ*), glyceraldehydes-3-phosphate dehydrogenase (*gapA*), DNA gyrase B subunit (*gyrB*), actin-like cytoskeleton protein (*mreB*), uridylate kinase (*pyrH*), recombination repair protein (*recA*) and RNA polymerase alpha-subunit (*rpoA*) was carried out as described previously [25, 26]. PCR amplicons were edited and assembled into consensus sequences using CLUSTAL W [22] (accession numbers are listed in S1 Table). DNA-DNA hybridization experiments using photobiotin-labelled DNA was performed at 44°C according to the method of Suzuki et al. [27]. The G+C content of the DNA was determined by high-performance liquid chromatography (HPLC) [28] as mean percentage values ± SD of triplicate measurements. DNA-DNA hybridization between *Vibrio* sp. nov. JCM 31412^T^, *V*. *brasiliensis* LMG 20546^T^, *V*. *nigripulchritudo* LMG 03896^T^, *V*. *caribbeanicus* ATCC BAA-2122^T^, *V*. *nereis* LMG 3895^T^ and *V*. *xuii* LMG 21346^T^ were performed in quadruplicate.

## Results and discussion

### Morphological description

**Fig 1:**
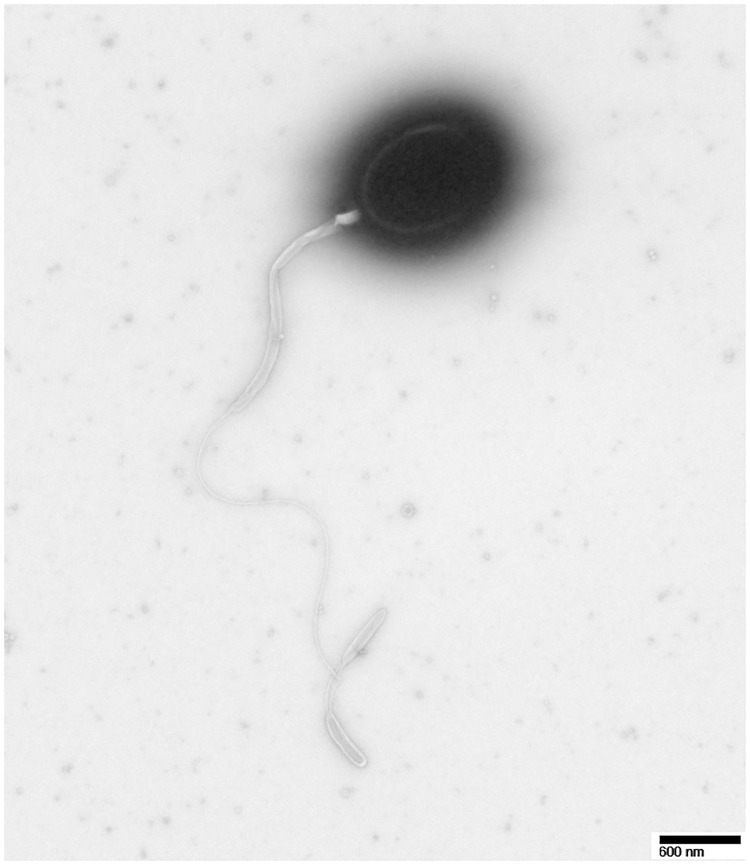
Negatively-stained transmission electron micrograph of *Vibrio japonicus* JCM 31412^T^ showing the sheathed single polar flagellum. Transmission electron microscopy of strain JCM 31412^T^ using H-7600 (Hitachi) at an operating voltage of 100kV. The micrograph shown was captured at a magnification of ×28,000. The bar indicates 600 nm.

Cells of strain JCM 31412^T^ were found to be a motile but swarming, ovoid and variable in size, ranging from 0.95–1.3 μm long and 0.6–0.9 μm wide. A sheathed single polar flagellum approximately 5.9 μm long was observed in TEM observations (Fig 1). Short rod bodies formed in older cultures. No endospores were observed. Colonies on ZoBell 2216E agar supplemented with 2.0% NaCl (w/v) were Gram-negative, cream colored, non-pigmented, non-luminescent, circular, smooth and convex, and 1–2 mm in diameter after incubation at 27°C for 24h.

### Phenotypic analysis

Phenotypic traits and characteristics of strain JCM 31412^T^ and closely related *Vibrio* species are listed in Table 1. Strain JCM 31412^T^ showed distinct phenotypic features of the genus *Vibrio* that were Gram-negative, oxidase positive, grown on TCBS agar and were facultative anaerobes [29]. It was further identified as a facultative anaerobe capable of both fermentative and respiratory metabolism. Furthermore, it required salt (0.5–9.0%), and was capable of growth from 10 to 37°C and at pH 7.0–12.0. Vigorous growth also occurred on ZoBell marine agar 2216E (Difco) and tryptic soy agar supplemented with 2.0% NaCl (w/v) at 25–37°C for 12 h. Optimal growth was observed at 35°C with 2.0% NaCl (w/v) at pH 8.0. Growth on TCBS agar (Nippon Beckton Dickinson) was also observed in the form of a yellow, convex, round colony, about 1 mm in diameter, after 72 h incubation at 28°C. Strain JCM 31412^T^ was positive for oxidase but negative for catalase and gelatinase activity. It was arginine dihydrolase (ADH)-positive, lysine decarboxylase (LDC)-positive and ornithine decarboxylase (ODC)-negative. Overall, the novel strain could be differentiated from phylogenetically known *Vibrio* species by several phenotypic characteristics.

### Antibiotic susceptibly test

Strain JCM 31412^T^ was susceptible to ampicillin (10 μg), ampicillin-sulbactam (10/10 μg), amoxicillin-clavulanic acid (20/10 μg), piperacillin (100 μg), piperacillin-tazobactam (100/10 μg), cefazolin (30 μg), cefepime (30 μg), cefotaxime (30 μg), cefoxitin (30 μg), ceftazidime (30 μg), cefuroxime sodium (parenteral) (30 μg), imipenem (10 μg), meropenem (10 μg), amikacin (30 μg), gentamicin (10 μg), tetracycline (30 μg), ciprofloxacin (5 μg), levofloxacin (5 μg), ofloxacin (5 μg) and trimethoprim-sulfamethoxazole (1.25/23.75 μg). In addition, susceptibility to vibriostatic agent O/129 was observed at both 10 and 150 μg per disc.

### Fatty acid analysis

The total cellular fatty acid composition of the strain JCM 31412^T^ and 3 other *Vibrio* species are shown in Table 2. The predominant fatty acids were C_12:0_, C_12:0_3OH, C_14:0_, C_16:0_, 3OH/iso-C_15:0_, C_17:1_ω8c, C_17:0_, summed isoH-C_15:1_ and 3OH-C_13:0_, summed ALDE-C_12:0_, unknown 10.928, isoI-C_16:1_ and C_14:0_3OH, summed C_16:1_ω7c and C_16:1_ω6c, and summed C_18:1_ω7c and C_18:1_ω6c, constituting 90.5% of the total cellular fatty acids. Forty-one fatty acids with 11–20 carbon atoms were detected. The fatty acid profile from strain JCM 31412^T^ showed significant differences from those of related type strains. Strain JCM 31412^T^ contained C_11:0_-3OH and C_20:1_ω7c which were not detected in other reference strains.

**Table 2:** Fatty acid composition (%) of *Vibrio* sp. nov. JCM 31412^T^ and 3 related *Vibrio* species.

Fatty acid	1	2	3	4
11:0	0.1			0.7
11:0-3OH	0.1			
12:0	3.8	3.4		15.9
14:0	4.5	7.8	8.7	2.8
16:0	21.9	11.4	18.7	9.5
17:0	1.5	0.2	0.2	0.8
12:0-3OH	4.3	0.8		0.8
13:0 iso	0.7	0.4		6.3
14:0 iso	4.5	7.8	8.7	2.8
15:0 iso	0.2	0.3		1.4
15:0 iso-3OH	1.2	0.1		0.9
16:0 iso	0.6	4.5	0.4	1.1
16:1 ω7c alcohol	0.3	0.3	0.3	1.8
16:1 ω9c	0.4	0.8	1.1	0.3
17:0 iso	0.2	0.1		1.6
20:1 ω7c	0.1			
17:1 ω6c	0.9	0.2	0.1	1.4
17:1 ω8c	1.9	0.4	0.4	1.8
Summed feature1^a^	1.2			0.4
Summed feature2^b^	6.4	1.8	5.0	2.6
Summed feature3^c^	27.7	44.0	48.7	27.4
Summed feature8^d^	17.3	15.9	12.7	8.0

Strains: 1, *Vibrio* sp. nov. JCM 31412^T^; 2, *Vibrio brasiliensis* LMG 20546^T^; 3, *Vibrio nigripulchritudo* LMG 03896^T^; 4, *Vibrio caribbeanicus* ATCC BAA-2122^T^. All data were obtained in this study.

^a^C15:1 iso H and/or C13:0-3OH

^b^C12:0 ALDE, unknown 10.928

^c^C16:1 ω7c and/or C16:1 ω6c

^d^C18:1 ω7c and/or C18:1 ω6c

### 16S rDNA genotypic analysis

**Fig 2:**
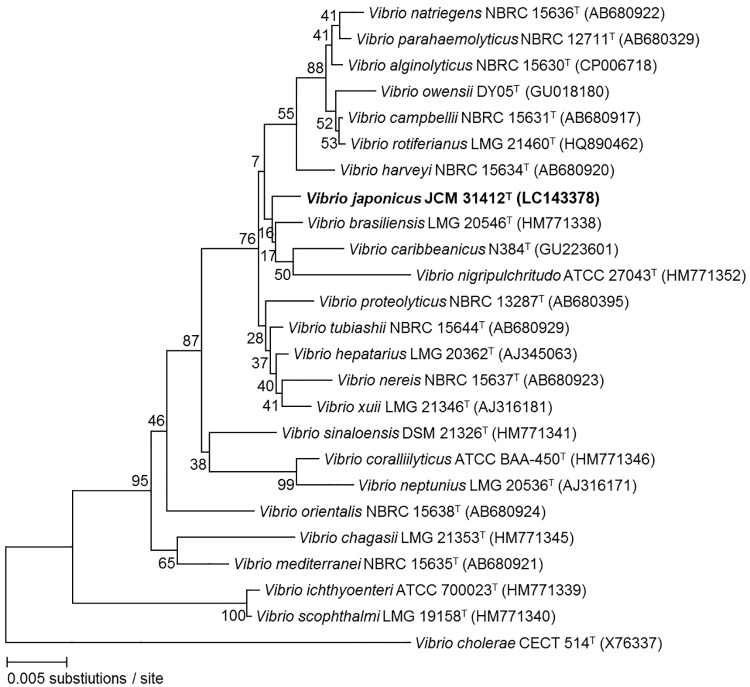
Neighboor-joining tree derived from 16S rDNA gene sequences showing the relationships between *Vibrio japonicus* JCM 31412^T^ and 24 other species. Numbers at the nodes show the percentage of bootstrap values. Bootstrap values shown are based on analysis of 100 replicates. *Bar* 0.005 substitutions per nucleotide position.

After analyzing and assembling the 1477-bp 16S rDNA gene sequence of strain JCM 31412^T^, the consensus sequence was used to query the GenBank database of NCBI using BLAST to identify strains with the highest sequence identity. Sequence searches demonstrated that strain JCM 31412^T^ belongs to the genus *Vibrio*. Analysis (query coverage 100%) further revealed that type strains of *V*. *brasiliensis* (LMG 20546^T^), *V*. *harveyi* (NBRC 13287^T^), *V*. *proteolyticus* (NBRC 13287^T^) and *V*. *carribeanicus* (ATCC BAA-2122^T^) were most closely related, sharing 98.2, 98.2, 97.8 and 97.8% 16S rDNA gene sequence identity, respectively. Moreover, a neighbor-joining tree derived from 16S rDNA gene sequences of 24 different *Vibrio* species including strain JCM 31412^T^ included novel strain JCM 31412^T^ in a cluster with *V*. *brasiliensis* LMG 20546^T^, *V*. *nigripulchritudo* ATCC 27043^T^ and *V*. *caribbeanicus* N384^T^ (Fig 2).

### MLSA

Vibrios are comprised of closely related species and are therefore difficult to identify [30]. MLSA of housekeeping genes has proven to be an accurate tool for delineation of microorganisms [31], as well as phylogenetic studies of the genus *Vibrio* [25]. To further optimize the taxonomic resolution, a neighbor-joining tree was therefore constructed from concatenated sequences of eight housekeeping genes (*topA*, *ftsZ*, *gapA*, *gyrB*, *mreB*, *pyrH*, *recA* and *rpoA*) as recommended by Sawabe et al. [32]. As a result, MLSA clustered strain JCM 31412^T^ in the Nereis clade along with *V*. *nereis* LMG 3895^T^ and *V*. *xuii* LMG 21346^T^, with high bootstrap values. Based on 16S rDNA gene sequence identity, the closest related neighbors were found to be type strains of *V*. *brasiliensis* (Orientalis clade), *V*. *nigripulchritudo* (Nigripulchritudo clade) and *V*. *carribeanicus* (Pectenicida clade). Phylogenic analysis of sequences of protein coding genes therefore revealed substantial differences from the phylogenic tree obtained based on 16S rDNA gene sequences due to low interspecies resolution with the latter [26, 33–35]. GenBank/EMBL/DDBJ accession numbers of the 16S rDNA, *topA*, *ftsZ*, *gapA*, *gyrB*, *mreB*, *pyrH*, *recA* and *rpoA* gene sequences of strain JCM 31412^T^, and all other gene sequences used in this study are listed in the S1 Table.

### DNA-DNA hybridization analysis

**Fig 3:**
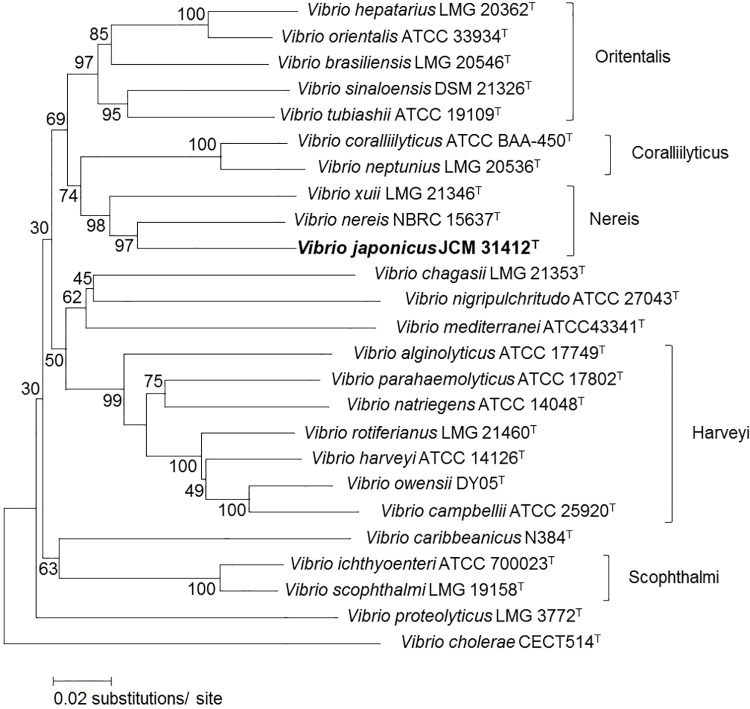
Neighbor-joining tree derived from concatenated sequences of *topA*, *ftsZ*, *gapA*, *gyrB*, *mreB*, *pyrH*, *recA* and *rpoA* genes (total length 5535 bp) showing the relationship between *Vibrio japonicus* JCM 31412^T^ and 24 related species. Numbers at the nodes show the percentage of bootstrap values. Bootstrap values shown are based on analysis of 100 replicates. *Bar* 0.02 substitutions per nucleotide position.

The phylogenetic tree based on 16S rDNA gene sequences of strain JCM 31412^T^ and related species showed that strain JCM 31412^T^ formed its closest phylogenetic cluster with type strains of *V*. *brasiliensis*, *V*. *nigripulchritudo* and *V*. *caribbeanicus* but with low bootstrap support (Fig 2). In contrast, MLSA revealed that strain JCM 31412^T^ formed a distinct cluster in the Nereis clade with type strains of *V*. *nereis* and *V*. *xuii*, with high bootstrap values (Fig 3). DNA-DNA hybridization experiments were therefore performed between novel strain JCM 31412^T^ and established strains grouped in the Nereis clade as well as closest phylogenetic neighbors based on 16S rDNA gene sequences. DNA of strain JCM 31412^T^ showed relatively low DNA-DNA relatedness with *V*. *brasiliensis* LMG 20546^T^ [15], *V*. *nigripulchritudo* LMG 03896^T^ [16], *V*. *caribbeanicus* ATCC BAA-2122^T^ [17], *V*. *nereis* LMG 3895^T^ [36] and *V*. *xuii* LMG 21346^T^ [15] (36.4±1.6, 24.8±1.8, 24.5±3.0, 36.4±2.6 and 32.7±3.4%, respectively), significantly below the recommended cut-off threshold of 70% DNA-DNA hybridization for the identification of bacterial species (Table 3) [37]. These results further support the suggestion that strain JCM 31412^T^ represents a species distinct from either of its nearest neighbors based on MLSA, as well as from type strains of *V*. *nereis* and *V*. *xuii*.

**Table 3:** DNA-DNA similarity between *Vibrio* sp. nov. JCM 31412^T^ and 5 related *Vibrio* species.

	1	2	3	4	5	6
*Vibrio* sp. nov. JCM 31412^T^	**100**					
*Vibrio brasiliensis* LMG 20546^T^	36.4±1.6	**100**				
*Vibrio nigripulchritudo* LMG 03896^T^	24.8±1.8	25.0±1.9	**100**			
*Vibrio caribbeanicus* ATCC BAA-2122^T^	24.5±3.0	27.0±2.8	23.0±2.1	**100**		
*Vibrio nereis* LMG 3895^T^	36.4±2.6	30.6±1.8	25.0±1.6	24.2±1.0	**100**	
*Vibrio xuii* LMG 21346^T^	32.7±3.4	28.3±3.6	24.5±4.1	19.4±0.4	44.5±3.8	**100**

Strains: 1, *Vibrio* sp. nov. JCM 31412^T^; 2, *Vibrio brasiliensis* LMG 20546^T^; 3, *Vibrio nigripulchritudo* LMG 03896^T^; 4, *Vibrio caribbeanicus* ATCC BAA-2122^T^; 5, *Vibrio nereis* LMG 3895^T^; 6, *Vibrio xuii* LMG 21346^T^. Values are expressed as means ± SD (n = 4).

### G+C content analysis

The mean G+C content of strain JCM 31412^T^ was calculated as 46.8 mol%, which falls within the range of 38–51 mol% previously found for other members of the genus *Vibrio* [38].

### Nucleotide sequence accession numbers

All sequence files are available on the GenBank nucleotide database with accession numbers for the 16S rDNA gene, *topA*, *ftsZ*, *gapA*, *gyrB*, *mreB*, *pyrH*, *recA* and *rpoA* sequences, respectively. Other relevant data are provided in S1 Table.

## Conclusion

This study characterized strain JCM 31412^T^ both phenotypically and genotypically, supporting its description as a novel and previously uncharacterized *Vibrio* species. Strain JCM 31412^T^ formed a stable group in the Nereis clade, and could be differentiated from closely related species based on phylogenetic analysis, DNA-DNA relatedness and phenotypic characterization. Overall, these data confirm that strain JCM 31412^T^ is a novel species belonging to the genus *Vibrio*, for which the name *Vibrio japonicus* sp. nov. is proposed.

## Description of *Vibrio japonicus* sp. nov.

*Vibrio japonicus* (ja.po’ni.cus N.L. masc. adj. *japonicus* pertaining to Japan, from where the type strain was originally isolated).

Cells ovoid (approx. 0.8 μm wide and 1.0μm long), Gram-negative, motile coccus with one flagellum. Flagellum sheaths observed. Grows well on ZoBell marine agar 2216E (Difco) and Tryptic soy agar supplemented with 2.0% NaCl (w/v) at 25–37°C for 12h. Colonies cream colored, translucent, smooth, rounded, with a diameter of 1–2 mm on ZoBell marine agar 2216E (Difco), motile but swarming. Growth at 10–37°C, NaCl concentrations of 0.5–9.0% and pH 7.0–12.0. Optimal growth at 35°C with 2.0% NaCl (w/v) at pH 8.0. Colonies yellow, convex, round and approximately 1 mm on TCBS agar after 72 h incubation at 28°C. Bioluminescence not observed. Susceptible to ampicillin (10 μg), ampicillin-sulbactam (10/10 μg), amoxicillin-clavulanic acid (20/10 μg), piperacillin (100 μg), piperacillin-tazobactam (100/10 μg), cefazolin (30 μg), cefepime (30 μg), cefotaxime (30 μg), cefoxitin (30 μg), ceftazidime (30 μg), cefuroxime sodium (parenteral) (30 μg), imipenem (10 μg), meropenem (10 μg), amikacin (30 μg), gentamicin (10 μg), tetracycline (30 μg), ciprofloxacin (5 μg), levofloxacin (5 μg), ofloxacin (5 μg) and trimethoprim-sulfamethoxazole (1.25/23.75 μg). Also susceptible to vibriostatic agent O/129 at both 10 and 150 μg. Catalase-negative, oxidase-positive, arginine dihydrolase-positive, lysine decarboxylase-positive and ornithine decarboxylase-negative. Also positive for alkaline phosphatase, esterase (C4), esterase lipase (C8), leucine arylamidase, valine arylamidase naphthol-As-BI-phosphohydrolase, α-galactosidase, β-glucuronidase tryptophan deaminase DNase and amylase activity (starch). Negative for lipase (C14), cystine arylamidase, trypsin, α-chymotrypsin, β-galactosidase, α-glucosidase, β-glucosidase, *N*-acetyl-β-glucosaminidase, α-mannosidase, α-fucosidase, ornithine decarboxylase and urease activity, hydrolysis of gelatin, casein and esculin. Positive for citrate utilization, production of indole, fermentation of glucose, sucrose, D-mannitol, melibiose and D-amygdaline, and assimilation of galactose, fructose, D-cellobiose, D-ribose, trehalose, D-raffinose, glycogen and *N*-acetylglucosamine. Negative for nitrate reduction to nitrate and H_2_S and acetoin (Voges-Proskauer) production. Able to utilize Dl-malic acid and sodium citrate but unable to utilize mannose, sorbose, D-lactose, L-rhamnose, D-arabinose, D-xylose, gentiobiose, D-turanose, D-tagatose, D-fucose, L-fucose, D-melezitose, amygdaline, inulin, salicin, arbutin, esculin, ferric citrate, xylitol, D-Arabitol, L-arabitol, glycerol, erythritol, adonitol, dulcitol, inositol, D-sorbitol, *N*-capric acid, adipic acid, gulconate, 2-keto-gluconate, 5-keto-gluconate, sodium pyruvate, phenyl acetate, sodium thiosulfate, methyl-αD-mannopyranoside, methyl-αD-glucopyranoside, methyl-β-D-xylopyranoside, *o*-nitrophenyl-β-D-galactopyranoside, *p*-nitrophenyl-β-D-galactopyranoside and urea. The most abundant fatty acids are C_12:0_, C_12:0_3OH, C_14:0_, C_16:0_, 3OH/iso-C_15:0_, C_17:1_ω8c, C_17:0_, summed isoH-C_15:1_ and 3OH-C_13:0_, summed ALDE-C_12:0_, unknown 10.928, isoI-C_16:1_ and C_14:0_3OH, summed C_16:1_ω7c and C_16:1_ω6c, and summed C_18:1_ω7c and C_18:1_ω6c. G+C content 46.8±0.1 mol%.

The type strain JCM 31412^T^ (= LMG 29636^T^ = ATCC TSD-62^T^) was isolated from seawater collected from the Inland Sea, (Setonaikai) Japan. GenBank/EMBL/DDBJ accession numbers for 16S rDNA, *topA*, *ftsZ*, *gapA*, *mreB*, *pyrH*, *recA* and *rpoA* gene sequences of strain JCM 31412^T^ are LC143378, LC143379, LC143380, LC143381, LC143382, LC143383, LC143384, LC143385 and LC143386, respectively.

## Supporting information

S1 Table GenBank accession numbers of 16S rDNA and housekeeping genes of *V*. *japonicus* JCM 31412^T^ sp. nov. and reference type strains. (DOCX)
