# Supplementary material for: Vibrio japonicus sp. nov., a novel member of the Nereis clade in the genus Vibrio isolated from the coast of Japan
Source: PLoS One. 2017 Feb 23;12(2):e0172164. doi: 10.1371/journal.pone.0172164 (PMC5322892; doi:10.1371/journal.pone.0172164)
Supplement: S1 Table — (DOCX) [file pone.0172164.s001.docx]

**S1 Table.** GenBank accession numbers of 16S rDNA and housekeeping genes of *V. japonicus* JCM 31412^T^ sp. nov. and reference type strains.

| **Strain** | **16S rDNA** | ***topA*** | ***ftsZ*** | ***gapA*** | ***gyrB*** | ***mreB*** | ***pyrH*** | ***recA*** | ***rpoA*** |
| --- | --- | --- | --- | --- | --- | --- | --- | --- | --- |
| *V. japonicus* JCM 31412^T^ | LC143378 | LC143379 | LC143380 | LC143381 | LC143382 | LC143383 | LC143384 | LC143385 | LC143386 |
| *V. brasiliensis* LMG 20546^T^ | HM771338 | HM771333 | HM771354 | HM771359 | HM771364 | HM771369 | HM771374 | HM771379 | HM771384 |
| *V. nigripulchritudo* ATCC 27043^T^ | HM771352 | DQ907505 | EF027347 | DQ907297 | AB298236 | DQ907437 | GU266290 | AJ842480 | AJ842667 |
| *V. caribbeanicus* N384^T^ | GU223601 | HM771337 | HM771358 | HM771363 | HM771368 | HM771373 | HM771378 | HM771383 | HM771388 |
| *V. ichthyoenteri* ATCC 700023^T^ | HM771339 | HM771334 | HM771355 | HM771360 | HM771365 | HM771370 | HM771375 | HM771380 | HM771385 |
| *V. scophthalmi* LMG 19158^T^ | HM771340 | HM771335 | HM771356 | HM771361 | HM771366 | HM771371 | HM771376 | HM771381 | HM771386 |
| *V. sinaloensis* DSM 21326^T^ | HM771341 | HM771336 | HM771357 | HM771362 | HM771367 | HM771372 | HM771377 | HM771382 | HM771387 |
| *V. harveyi* ATCC 14126^T^ | AB680920 | DQ907488 | DQ907350 | DQ449616 | DQ648280 | DQ907422 | EU118238 | AJ842440 | AJ842627 |
| *V. natriegens* ATCC 14048^T^ | AB680922 | DQ907500 | DQ907359 | DQ907294 | AB298232 | DQ907432 | FM202573 | AJ842473 | AJ842658 |
| *V. campbellii* ATCC25920^T^ | AB680917 | EF596698 | EF596542 | EF596565 | EF596590 | EF596615 | EF596641 | EF596670 | AJ842564 |
| *V. chagasii* LMG 21353^T^ | HM771345 | DQ481649 | DQ996590 | DQ481611 | AM162568 | DQ481637 | EU118252 | AJ842385 | AJ842572 |
| *V. coralliilyticus* ATCC BAA-450^T^ | HM771346 | EF114213 | DQ907341 | DQ907279 | AB298210 | DQ907412 | GU266292 | AJ842402 | AJ842587 |
| *V. parahaemolyticus* ATCC 17802^T^ | AB680329 | DQ907509 | DQ907367 | DQ449618 | AF007287 | DQ907440 | EU118240 | AJ842490 | AJ842677 |
| *V. alginolyticus* ATCC 43341^T^ | CP006718 | DQ907472 | EF027344 | DQ907274 | AB298202 | DQ907405 | GU266285 | AJ842373 | AJ842558 |
| *V. mediterranei* ATCC 43341^T^ | AB680921 | DQ907495 | DQ907356 | DQ907290 | AB298228 | DQ907428 | GU266288 | AJ842459 | AJ842644 |
| *V. neptunius* LMG 20536^T^ | AJ316171 | DQ907503 | DQ907361 | DQ907296 | AB298234 | DQ907435 | GU266291 | AJ842478 | AJ842665 |
| *V. tubiashii* ATCC 19109^T^ | AB680929 | DQ907521 | DQ907381 | DQ907312 | AB298251 | DQ907453 | JF316670 | AJ842518 | AJ842734 |
| *V. orientalis* ATCC 33934^T^ | AB680924 | DQ907507 | DQ907365 | EU130488 | EF380260 | DQ907439 | EU118243 | AJ842485 | AJ842672 |
| *V. hepatarius* LMG 20362^T^ | AJ345063 | DQ907491 | DQ907352 | DQ907285 | AB298222 | DQ907424 | JF316674 | AJ842444 | AJ842631 |
| *V. rotiferianus* LMG 21460^T^ | HQ890462 | DQ907515 | DQ907372 | DQ449619 | EU118210 | DQ907445 | EF596722 | AJ842501 | AJ842688 |
| *V. proteolyticus* LMG 3772^T^ | AB680395 | DQ907514 | EF114210 | DQ907305 | AB298261 | DQ907444 | NA | AJ842499 | AJ842686 |
| *V. xuii* LMG 21346^T^ | AJ316181 | DQ907524 | DQ907384 | DQ907315 | AB298254 | DQ907456 | GU266284 | AJ842529 | AJ842742 |
| *V. owensii* DY05^T^ | GU018180 | GU111255 | GU111257 | HE653272 | HE653273 | GU111259 | GU111253 | KF899345 | GU111250 |
| *V. nereis* NBRC 15637^T^ | AB680923 | DQ907504 | DQ907362 | DQ449617 | AB298235 | DQ907436 | JN968379 | AJ580870 | AJ842666 |
| *V. cholerae* CECT 514^T^ | X76337 | HE805631 | HE805627 | HE805629 | FM202624 | NA | FM202582 | AM942078 | HE805630 |

NA: not available
